# PET-Based Human Dosimetry of ^68^Ga-NODAGA-Exendin-4, a Tracer for β-Cell Imaging

**DOI:** 10.2967/jnumed.119.228627

**Published:** 2020-01

**Authors:** Marti Boss, Mijke Buitinga, Tom J.P. Jansen, Maarten Brom, Eric P. Visser, Martin Gotthardt

**Affiliations:** Department of Radiology and Nuclear Medicine, Radboud University Medical Center, Nijmegen, The Netherlands

**Keywords:** dosimetry, exendin-4, β-cells, insulinoma, congenital hyperinsulinism

## Abstract

^68^Ga-NODAGA-exendin-4 is a promising tracer for β-cell imaging using PET/CT. Possible applications include preoperative visualization of insulinomas and discrimination between focal and diffuse forms of congenital hyperinsulinism. There is also a significant role for this tracer in extending our knowledge on the role of β-cell mass in the pathophysiology of type 1 and type 2 diabetes by enabling noninvasive quantification of tracer uptake as a measure for β-cell mass. Calculating radiation doses from this tracer is important to assess its safety for use in patients (including young children) with benign diseases and healthy individuals. **Methods:** Six patients with hyperinsulinemic hypoglycemia were included. After intravenous injection of 100 MBq of the tracer, 4 successive PET/CT scans were obtained at 30, 60, 120, and 240 min after injection. Tracer activity in the pancreas, kidneys, duodenum, and remainder of the body were determined, and time-integrated activity coefficients for the measured organs were calculated. OLINDA/EXM software, version 1.1, was applied to calculate radiation doses using the reference adult male and female models and to estimate radiation doses to children. **Results:** The mean total effective dose for adults was very low (0.71 ± 0.07 mSv for a standard injected dose of 100 MBq). The organ with the highest absorbed dose was the kidney (47.3 ± 10.2 mGy/100 MBq). The estimated effective dose was 2.32 ± 0.32 mSv for an injected dose of 20 MBq in newborns. This dose decreased to 0.77 ± 0.11 mSv/20 MBq for 1-y-old children and 0.59 ± 0.05 mSv for an injected dose of 30 MBq in 5-y-old children. **Conclusion:** Our human PET/CT-based dosimetric calculations show that the effective radiation doses from the novel tracer ^68^Ga-NODAGA-exendin-4 are very low for adults and children. The doses are lower than reported for other polypeptide tracers such as somatostatin analogs (2.1–2.6 mSv/100 MBq) and are beneficial for application as a research tool, especially when repeated examinations are needed.

For new radiopharmaceuticals, estimating effective radiation doses is important to determine safety for patients. The radiopharmaceutical ^68^Ga-NODAGA-exendin-4 binds specifically to the glucagonlike peptide 1 receptor, which is expressed on pancreatic β-cells (1). It is a promising tracer for in vivo targeting of β-cells using PET/CT, with several possible applications in clinical diagnostics for insulin-producing lesions and in diabetes research. Since these applications entail the use of this radiopharmaceutical in patients with a generally normal life expectancy (i.e., patients with benign tumors or diabetes mellitus) and even in completely healthy individuals in control groups for research, performing dosimetric calculations is of substantial importance.

A promising application of this new tracer is preoperative visualization of insulin-producing neuroendocrine tumors (insulinomas), for which current standard imaging methods have limited sensitivity (2–4). Clinical studies have shown the potential of ^111^In-labeled exendin for detection of such tumors with SPECT/CT (5–7). ^68^Ga-exendin PET/CT has been demonstrated to be superior to ^111^In-exendin SPECT/CT in a small crossover clinical trial (8), and its superiority over conventional imaging was shown in a large prospective trial (9). Currently, an ongoing multicenter prospective trial (NCT03189953) is comparing the sensitivity of ^68^Ga-NODAGA-exendin-4 PET/CT for detection of insulinomas with all current standard noninvasive imaging modalities (somatostatin receptor PET/CT, triple-phase CT, and MRI). In addition, ^68^Ga-NODAGA-exendin-4 PET/CT is being investigated as a potential new imaging method to distinguish between diffuse and focal forms of congenital hyperinsulinism (NCT03768518) (10,11).

In addition to these clinical applications, ^68^Ga-NODAGA-exendin-4 has great potential as a research tool to determine β-cell mass in vivo. Changes in β-cell mass over time in type 1 and type 2 diabetes are not well characterized, since previous studies had to rely on data obtained after autopsy or pancreatectomy, which provide data at only a single point in time and do not always provide information from the complete pancreas (12,13). By using ^68^Ga-NODAGA-exendin-4 PET/CT, it would be possible to investigate the dynamics of β-cell mass during onset and progression of the disease. Hereby, this technique can contribute to knowledge about the role of β-cell mass in the pathophysiology of diabetes.

For these applications of ^68^Ga-NODAGA-exendin-4, a low effective radiation dose is important, especially if repeated imaging is a possibility. Moreover, low effective doses are essential since an important application involves use in children, often very young, with congenital hyperinsulinism. In this study, we therefore performed human PET-based dosimetry on adults with suspected insulinoma to determine effective radiation doses and organ-absorbed doses, and we extrapolated these data to estimate radiation doses to children.

## MATERIALS AND METHODS

### Radiopharmaceutical Preparation

Hydrochloric acid was purchased from Rotem Industries. All other components were purchased as a single disposable kit (reagent and hardware kit for synthesis of ^68^Ga peptides using cationic purification; ABX). ^68^Ga-NODAGA-exendin-4 was manufactured using a synthesizer module (GRP synthesizer; Scintomics). ^68^Ga-chloride was obtained from a ^68^Ge/^68^Ga generator (Galliapharm; Eckert and Ziegler). The generator was eluted with 10 mL of 0.1 M hydrochloric acid, and ^68^Ga was trapped on a polystyrene-H+ cartridge. The cartridge was eluted with 1.5 mL of 5 M NaCl in 0.1 M hydrochloric acid into the reaction vial containing 200 μL of exendin-NODAGA (10 μg of peptide in water for injection), 475 μL of 2.5 M 4-(2-hydroxyethyl)-1-piperazineethanesulfonic acid buffer, and 50 μL of ascorbic acid (100 mg/mL) in water for injection. After 15 min of incubation at 100°C, the vessel was cooled and 2 mL of 50 mM ethylenediaminetetraacetic acid/0.15% polysorbate 80 were added. ^68^Ga-NODAGA-exendin-4 was purified on a hydrophilic–lipophilic balance cartridge and sterilized by being passed through a 0.2-μm filter (Millex GV).

### Human Subjects

Six adults with suspected insulinoma based on biochemically confirmed hyperinsulinemic hypoglycemia were included. The hypoglycemia was diagnosed by positive results on a fasting test, with the occurrence of neuroglycopenic symptoms in the fasting state combined with inappropriately low plasma glucose levels and high insulin and C-peptide levels. Table 1 shows the characteristics of the patients, who were included as part of an ongoing prospective clinical trial (NCT03189953) for evaluation of ^68^Ga-NODAGA-exendin-4 for diagnosis of insulinoma. Lesions were detected in 3 of the included patients (Supplemental Fig. 1; supplemental materials are available at http://jnm.snmjournals.org). They underwent surgery, which confirmed the presence of lesions at the indicated sites. These lesions were histopathologically confirmed to be insulinomas. In the other 3 patients, nesidioblastosis was suspected, but no definite diagnosis could be reached. The study was approved by the Radboud University Medical Center institutional review board, and all participants provided written informed consent.

**TABLE 1:** Patient Characteristics, Injected Activities, and Time After Injection of Performed PET/CT Scans

					Time after injection (min)			
Patient no.	Sex	Age (y)	Weight (kg)	Injected activity (MBq)	Scan 1	Scan 2	Scan 3	Scan 4
1	F	24	72.0	106.1	32	66	121	239
2	F	53	58.0	107.5	32	62	120	238
3	F	55	75.6	108.0	30	83	119	235
4	M	65	83.6	105.0	30	61	120	234
5	F	64	88.8	105.1	30	56	115	235
6	M	63	84.5	101.6	30	62	118	236

### PET/CT Acquisition

Imaging was performed on a Siemens Biograph mCT-40 time-of-flight PET/CT scanner. The patients were injected intravenously with ^68^Ga-NODAGA-exendin-4 (105.6 ± 2.3 MBq; peptide dose, 4–7 μg). Four consecutive PET scans were obtained at 30, 60, 120, and 240 min after injection. Images were acquired at 2 bed positions that included the liver, pancreas, and kidneys, at 10 min per bed position. A low-dose CT scan without contrast medium (40 mAs and 130 kV) was acquired for anatomic localization and attenuation correction. The size of the CT transaxial matrix was 512 × 512 (0.98 × 0.98 mm), and the CT slice width was 3 mm. High-definition reconstruction of the images was performed with 3 iterations, 21 subsets, and a postreconstruction gaussian filter of 3 mm in full width at half maximum. The transaxial PET matrix size was 256 × 256, and the pixel size was 3.18 × 3.18 × 3 mm.

### Image Analysis

The PET/CT images were analyzed using Inveon Research Workplace software (version 4.1; Siemens Healthcare). Volumes of interest were drawn over the kidneys, pancreas, and duodenum, which showed visually identifiable tracer uptake on the PET/CT images. The total activity in each organ at each time point was determined by multiplying the mean activity concentration (Bq/mL) by the CT-derived volume of interest. Since the uptake in the kidneys is high compared with the pancreas, there is spillover of activity in the left kidney to the pancreatic tail, which could lead to an overestimation of tracer uptake in the pancreatic tail. To correct for this overestimation, the volume of interest of the left kidney was dilated by 9 mm and subtracted from the region of interest of the pancreas. The tracer uptake in this part of the pancreas was then assumed to equal the mean uptake in the rest of the pancreas. Organ time–activity curves were plotted, and cumulated activity was calculated as the area under the curve by the trapezoid rule. Only physical decay was assumed after the last measurement. The time-integrated activity coefficient was determined by dividing the cumulated activity by the total injected activity. The activity in the remainder of the body was determined by multiplying the total activity in the scanned area, minus the source organs, by the total-body volume, based on the height and body weight of the patient. With this determination, a homogeneous tracer distribution over the remainder of the body was assumed.

### Radiation Dose Estimates

The calculated time–activity coefficients of the source organs and the remainder of the body were used as input in OLINDA/EXM software, version 1.1. Organ-absorbed doses and total effective doses for each patient were obtained using the reference adult male and female models. To estimate radiation doses in children, a comparable biodistribution of the tracer between adults and children was assumed. Organ-absorbed doses and effective doses were determined using the newborn, 1-y-old, and 5-y-old models.

## RESULTS

**FIGURE 1.**
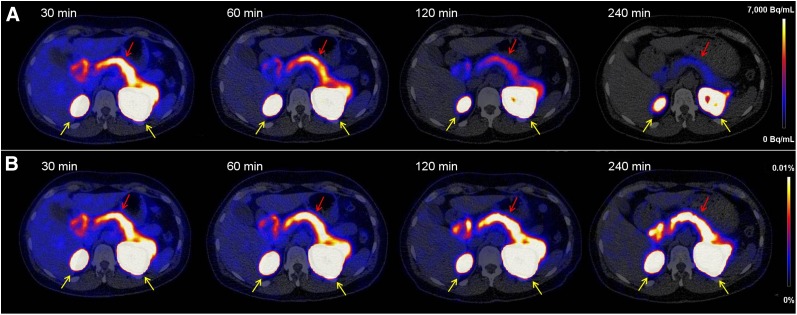
Transversal PET/CT images of abdomen showing biodistribution of ^68^Ga-NODAGA-exendin-4 at 30, 60, 120, and 240 min after injection. (A) Tracer accumulation in Bq/mL, showing washout as well as physical decay. (B) Tracer accumulation in percentage injected dose/g of tissue, showing only washout of tracer. Kidneys are indicated by yellow arrows and pancreas by red arrows.

**FIGURE 2.**
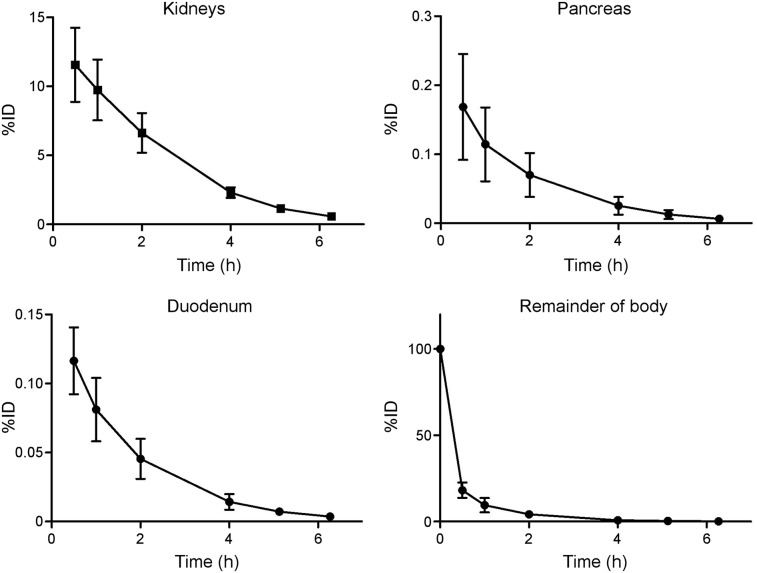
Time–activity curves of kidneys, pancreas, duodenum, and remainder of body (*n* = 6). Data are mean ± SD. %ID = percentage injected dose.

Figure 1 shows typical axial slices of ^68^Ga-NODAGA-exendin-4 PET/CT images at various time points after injection. Uptake was highest in the kidneys because of renal clearance of the tracer. Furthermore, the highest specific tracer uptake was in the pancreas and proximal duodenum. Retention of the tracer in these organs over time was high (Fig. 1B). As a result, physical decay is the main cause of reduction in activity concentration in the organs over time (Fig. 1A). The mean time–activity curves for these organs and the remainder of the body for the patients are depicted in Figure 2. Table 2 lists the mean organ-absorbed doses and the effective dose acquired using the reference adult male and female models in OLINDA/EXM. The organ with the highest absorbed dose was the kidney (0.47 ± 0.10 mGy/MBq), followed by the pancreas (0.023 ± 0.008 mGy/MBq), the adrenals (0.012 ± 0.002 mGy/MBq), the spleen (0.011 ± 0.001 mGy/MBq), and the small intestine (0.008 ± 0.001 mGy/MBq). Doses to the pancreas and small intestine partly consist of the organ self-dose but will mostly originate from the kidneys. The mean total effective dose was very low (0.007 ± 0.0007 mSv/MBq).

**TABLE 2:** Organ-Absorbed Doses (mGy/MBq) and Effective Doses (mSv/MBq) Acquired Using Reference Adult Models in OLINDA/EXM 1.1

Site (*n* = 6)	Mean	SD
Adrenals	0.012	0.002
Brain	0.005	0.0006
Breasts	0.005	0.0006
Gallbladder wall	0.008	0.0008
Stomach wall	0.007	0.0007
Heart wall	0.006	0.0006
Kidneys	0.472	0.1019
Intestine, lower large: wall	0.006	0.0007
Intestine, upper large: wall	0.007	0.0007
Intestine, small	0.008	0.0010
Liver	0.008	0.0008
Lungs	0.006	0.0006
Muscle	0.006	0.0006
Ovaries	0.006	0.0008
Pancreas	0.023	0.0083
Red marrow	0.006	0.0005
Osteogenic cells	0.008	0.0010
Skin	0.005	0.0005
Spleen	0.001	0.0001
Testes	0.002	0.0003
Thymus	0.005	0.0006
Thyroid	0.005	0.0006
Urinary bladder wall	0.005	0.0007
Uterus	0.004	0.0003
Total body	0.008	0.0009
Effective dose (mSv/MBq)	0.007	0.0007

Estimated doses for newborn children and children 1 and 5 y old are depicted in Table 3. The kidney was the dose-limiting organ, receiving a dose of 5.4 ± 1.1 mGy/MBq in newborns. This dose decreased with age to 2.0 ± 0.4 mGy/MBq in 1-y-old children and 1.1 ± 0.2 mGy/MBq in 5-y-old children. The estimated effective dose for newborns was 0.12 ± 0.02 mSv/MBq. This low dose decreased further to 0.019 ± 0.003 mSv/MBq for 5-y-old children.

**TABLE 3:** Estimated Absorbed Doses (mGy/MBq) and Effective Doses (mSv/MBq) in Children Acquired Using Newborn, 1-Year-Old, and 5-Year-Old Models in OLINDA/EXM 1.1

Site	Newborn	1-y-old	5-y-old
Kidneys	5.430 ± 1.086	2.037 ± 0.408	1.125 ± 0.225
Pancreas	0.530 ± 0.217	0.160 ± 0.062	0.082 ± 0.021
Adrenals	0.129 ± 0.015	0.059 ± 0.007	0.033 ± 0.004
Spleen	0.112 ± 0.017	0.050 ± 0.006	0.028 ± 0.003
Small intestine	0.114 ± 0.017	0.047 ± 0.007	0.047 ± 0.007
Whole body	0.117 ± 0.014	0.046 ± 0.006	0.023 ± 0.003
Effective dose (mSv/MBq)	0.116 ± 0.016	0.038 ± 0.005	0.019 ± 0.003

Data are mean ± SD.

## DISCUSSION

Up to now, the reported dosimetry of ^68^Ga-labeled exendin has relied on extrapolation of human SPECT/CT scans using ^111^In-labeled exendin or data from small-animal SPECT with ^111^In-labeled exendin. In this study, the PET/CT-based dosimetry of ^68^Ga-NODAGA-exendin-4 was assessed for the first time, to our knowledge, using human PET/CT scans. The mean total-body effective dose was very low (0.71 ± 0.07 mSv/100 MBq, which is currently the standard injected dose in adults). The highest mean absorbed dose, 47.2 ± 10.2 mGy/100 MBq, was received by the kidneys. The calculated effective dose received by adults from ^68^Ga-NODAGA-exendin-4 was lower than total-body effective doses reported in dosimetry studies for the different tracers used for somatostatin receptor imaging—^68^Ga-DOTATOC, ^68^Ga-DOTANOC, and ^68^Ga-DOTATATE—which were between 2.1 and 2.6 mSv/100 MBq (14–17). The dose is also much lower than the dose from a low-dose CT scan, which is 4.5 mSv for a whole-body scan and 1.8 mSv for a scan of the abdomen at 1 bed position in adults. This low dose will allow repeated examinations using ^68^Ga-NODAGA-exendin-4 in adults. The low total-body effective dose allows 2–4 PET/CT examinations per year in adults (depending on the range of the low-dose CT scan) and up to 14 PET/MRI examinations without exceeding the maximum dose of 10 mSv. Even with this number of examinations, the kidney-absorbed dose will remain well below 7 Gy, which is the threshold for the human kidney acute dose (18). The absorbed dose to the red marrow was only 0.6 ± 0.05 mGy/100 MBq. Over 400 examinations could be performed while still remaining below 0.25 Gy per year, which has been shown to have no damaging effects (18). The number of annual examinations will therefore not be limited by the radiation dose to the kidneys or red marrow.

The dosimetry data derived from adult patients were extrapolated to children of various ages. The estimated total effective dose in newborns was 2.3 ± 0.3 mSv/20 MBq, which is the dose given to children with a body weight under 12.5 kg. This effective dose is significantly higher than in adults but still nearly 4-fold lower than the effective dose reported for the standard PET tracer currently used to diagnose congenital hyperinsulinism in newborns—^18^F-DOPA 6-^18^F-fluoro-l-3,4-dihydroxyphenylalanine—which is 0.4 ± 0.04 mSv/MBq, resulting in a total dose of 8 ± 0.8 mSv/20 MBq (19).

The pancreas was the organ receiving the second highest radiation dose (2.3 ± 0.8 mGy/100 MBq in adults). The absorbed dose to the pancreas was determined by assuming a homogeneous distribution of the radiotracer across the organ. However, since exendin specifically targets the β-cells in the islets of Langerhans in the pancreas, the activity concentration in these small clusters of endocrine cells is much higher than in exocrine pancreatic tissue (20). Therefore, the radiation dose to the islets may be underestimated using organ-based dosimetry, and the dose to the exocrine pancreas may be overestimated. This issue was previously examined using a macro- and small-scale dosimetry model combining animal and human data using ^111^In-labeled exendin. These data were extrapolated to ^68^Ga, showing that the absorbed dose to the islets of Langerhans (maximum, 66.0 mGy for ^111^In and 1.38 mGy for ^68^Ga) (21) remains clearly below the dose known to cause diabetes, which was estimated as a relative risk of 1:61 (95% confidence interval, 1:21–2:68) with a radiation dose of 1 Gy to the tail of the pancreas (22). The estimated time-integrated activity coefficients for the pancreas and the kidneys in this study were obtained by converting the ^111^In-exendin SPECT/CT scans of 5 humans to ^68^Ga. When comparing these to the current data, we found approximately 3 times higher time-integrated activity coefficients for the pancreas (0.0034 ± 0.0014 vs. 0.0012 ± 0.0001) and about 2 times lower coefficients for the kidneys (0.29 ± 0.05 vs. 0.5 ± 0.05). Because of the almost equal contribution of both these organs to the islet dose with ^68^Ga (21), these differences will not greatly increase the estimated absorbed dose to the islets. So, even though the data in this study show some differences from ^111^In-exendin, it remains clear that use of ^68^Ga-NODAGA-exendin-4 will not result in islet toxicity, especially since the estimated absorbed doses to the pancreas in this study (10.6 ± 4.5 mGy/20 MBq in newborns, 3.2 ± 1.2 mGy/20 MBq in 1-y-olds, 2.5 ± 0.4 mGy/30 MBq in 5-y-olds, and 2.3 ± 0.8 mGy/100 MBq in adults) are far lower than 1 Gy.

Previous studies on dosimetry for ^68^Ga-labeled exendin have relied on animal data extrapolated to humans. Extrapolation of organ and whole-body dosimetry data to other species might be unreliable because of variations in tracer biodistribution between species, resulting, for instance, from differences in receptor expression, such as the strikingly higher glucagonlike peptide 1 receptor expression found in the lungs and thyroid gland of rodents than in humans (23). Reported total effective doses vary considerably between the different animal models that have previously been used. Reported effective doses range from 0.012 to 0.032 mSv/MBq depending on the species studied (Sprague-Dawley rats, Lewis rats, pigs, cynomolgus monkeys, and 1 human) (24–26). Also, estimated organ-absorbed doses are not consistent. On the basis of a Rip1Tag2 mouse model of pancreatic β-cell tumors, an extrapolated human kidney-absorbed dose of 1.85 mGy/MBq was reported (25). In other studies, based on various different species, lower kidney doses were found, better corresponding to the kidney doses found in this study. In Sprague-Dawley rats, a kidney dose of 0.52 mGy/MBq was described (26), and in a dosimetry study comparing several species (Lewis rats, pigs, cynomolgus monkeys, and 1 human), similar kidney doses (0.25–0.65 mGy/MBq) were reported.

## CONCLUSION

Our dosimetric calculations demonstrate low effective radiation doses from ^68^Ga-NODAGA-exendin-4 to adults and children. These doses are severalfold lower than those reported for current tracers used for imaging of insulinomas or neuroendocrine tumors and congenital hyperinsulinism, and they are much lower than doses from a low-dose CT scan. These low doses are beneficial for the application of ^68^Ga-NODAGA-exendin-4 as a diagnostic or research tool in children, patients with benign diseases, and healthy volunteers, allowing repeated examinations in prospective follow-up studies, such as to examine β-cell mass in different phases of progression of diabetes mellitus or to assess transplantation success and monitor the survival of transplanted islets.

## DISCLOSURE

This work was supported by BetaCure (FP7/2014-2018, grant agreement 602812) and INNODIA (IMI2-JU, grant agreement 115797). Martin Gotthardt is an inventor and holder of the patent “Invention Affecting Glucagonlike Peptide 1 and Exendin” (Philipps-Universität Marburg, June 17, 2009). No other potential conflict of interest relevant to this article was reported.

## KEY POINTS

**QUESTION:** Do the absorbed and effective radiation doses from the novel tracer ^68^Ga-NODAGA-exendin-4 to adults and children benefit its use as a diagnostic and research tool?
**PERTINENT FINDINGS:** A human PET/CT-based dosimetry study on 6 patients imaged at 3 time points was performed. The mean total effective dose for adults and children was very low (0.71 ± 0.07 mSv for a standard injected dose of 100 MBq in adults; 2.32 ± 0.32 mSv for an injected dose of 20 MBq in newborns).
**IMPLICATIONS FOR PATIENT CARE:** ^68^Ga-NODAGA-exendin-4 can safely be applied as a diagnostic or research tool in children, patients with benign diseases, and healthy volunteers, even allowing for repeated examinations in prospective follow-up studies.
