# Supplementary material for: PET-Based Human Dosimetry of 68Ga-NODAGA-Exendin-4, a Tracer for β-Cell Imaging
Source: J Nucl Med. 2020 Jan;61(1):112–6. doi: 10.2967/jnumed.119.228627 (PMC6954461; doi:10.2967/jnumed.119.228627)
Supplement: Supplementary file 1 [file jnm228627SupplementaryData.pdf]

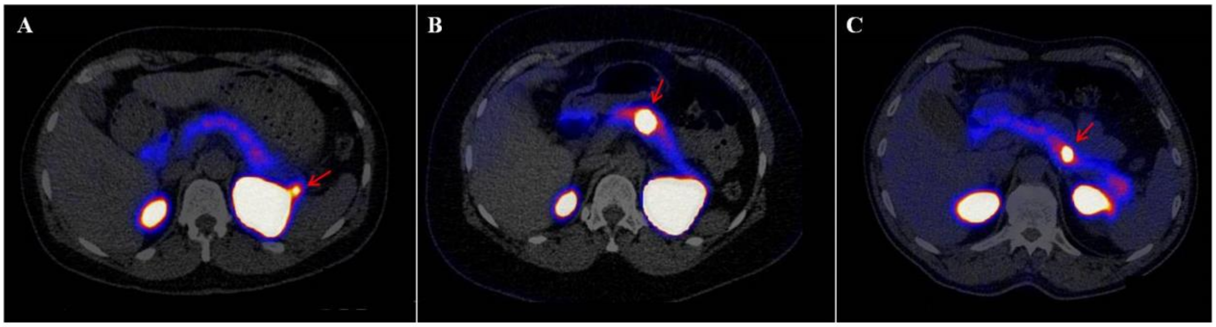

Supplemental figure 1: Transversal fused PET/CT images of the abdomen showing the detected insulinomas in patient 2 (A), patient 3 (B) and patient 4 (C). Lesions are indicated with red arrows.
